# Loss of prolyl hydroxylase domain protein 2 in vascular endothelium increases pericyte coverage and promotes pulmonary arterial remodeling

**DOI:** 10.18632/oncotarget.11585

**Published:** 2016-08-24

**Authors:** Shuo Wang, Heng Zeng, Xue-Jiao Xie, Yong-Kang Tao, Xiaochen He, Richard J. Roman, Judy L. Aschner, Jian-Xiong Chen

**Affiliations:** ^1^ Department of Pharmacology and Toxicology, University of Mississippi Medical Center, School of Medicine, Jackson, MS, USA; ^2^ School of Integrated Chinese and Western Medicine, Hunan University of Chinese Medicine, Changsha, Hunan, China; ^3^ Department of Pediatrics, Albert Einstein College of Medicine and The Children's Hospital at Montefiore, Bronx, NY, USA

**Keywords:** endothelial, prolyl hydroxylase-2, HIF, pulmonary hypertension, Pathology Section

## Abstract

Pulmonary arterial hypertension (PAH) is a leading cause of heart failure. Although pulmonary endothelial dysfunction plays a crucial role in the progression of the PAH, the underlying mechanisms are poorly understood. The HIF-α hydroxylase system is a key player in the regulation of vascular remodeling. Knockout of HIF-2α has been reported to cause pulmonary hypertension. The present study examined the role of endothelial cell specific prolyl hydroxylase-2 (PHD2) in the development of PAH and pulmonary vascular remodeling. The PHD2^f/f^ mouse was crossbred with VE-Cadherin-Cre promoter mouse to generate an endothelial specific PHD2 knockout (Cdh5-Cre-PHD2^EC^KO) mouse. Pulmonary arterial pressure and the size of the right ventricle was significantly elevated in the PHD2^EC^KO mice relative to the PHD2^f/f^ controls. Knockout of PHD2 in EC was associated with vascular remodeling, as evidenced by an increase in pulmonary arterial media to lumen ratio and number of muscularized arterioles. The pericyte coverage and vascular smooth muscle cells were also significantly increased in the PA. The increase in vascular pericytes was associated with elevated expression of fibroblast specific protein-1 (FSP-1). Moreover, perivascular interstitial fibrosis of pulmonary arteries was significantly increased in the PHD2^EC^KO mice. Mechanistically, knockout of PHD2 in EC increased the expression of Notch3 and transforming growth factor (TGF-β) in the lung tissue. We conclude that the expression of PHD2 in endothelial cells plays a critical role in preventing pulmonary arterial remodeling in mice. Increased Notch3/TGF-β signaling and excessive pericyte coverage may be contributing to the development of PAH following deletion of endothelial PHD2.

## INTRODUCTION

Pulmonary arterial hypertension (PAH), the leading cause of right heart failure, is associated with a high mortality rate. There is no cure for human PAH and approximately 20,000 people die annually of PAH in the United States [1]. PAH is characterized by structural remodeling of distal pulmonary arteries, causing vessel wall thickening and luminal occlusion by vascular smooth muscle and endothelial cell (EC) proliferation [2-4]. Clinically, PAH exhibits elevated pulmonary arterial pressure (PAP) and right ventricular hypertrophy [5]. Although it is well known that pulmonary microvascular remodeling plays a crucial role in the progression of the disease, the underlying mechanisms are not well defined. The endothelium plays a crucial role in vascular remodeling. Abnormalities in endothelial cell proliferation, differentiation and cross-talk to pericytes and vascular smooth muscle cells (VSMCs) are basic mechanisms underlying the pathogenesis of many cardiovascular diseases, including PAH and atherosclerosis. Despite increased knowledge about the role of endothelial dysfunction in the pathogenesis of PAH [2], how pulmonary endothelial dysfunction initiates pulmonary vascular remodeling and PAH remains unknown. A recent study highlighted the importance of endothelial cell/pericyte interactions in the pulmonary arterial remodeling in PAH. They found that pulmonary endothelial dysfunction disrupts FGF2/IL-6 signaling and increases microvascular pericyte coverage, which contributes to remodeling of small pulmonary arterioles in PAH [6].

Prolyl hydroxylase domain proteins (PHD) are the oxygen sensing molecules that form a complex von Hippel-Lindau protein (VHL) and degrade hypoxia inducible factor-α (HIF-α) [7-10]. There are at least three PHD isoforms in mammals, including PHD1, PHD2 and PHD3 [7, 11-13]. Based on its expression pattern and dominant effects under normoxic conditions, PHD2 is considered as the most important HIF-α-regulating isoform in the lung [14-16]. PHD2 is also essential during embryogenesis. Disruption of PHD2, but not PHD1 or PHD3, is lethal during embryonic development [17]. In pulmonary circulation, HIF-2α is strongly expressed in EC of the lung and is a key mediator in the development of pulmonary hypertension [18-20]. Studies in humans, suggest a role for the HIF-α hydroxylase system, particularly the PHD2/HIF-2α pathway, in the regulation of pulmonary vascular function in response to hypoxia [21-23]. In mice, activation of HIF-2α promotes the development of pulmonary hypertension and right ventricular hypertrophy [18]. In contrast, heterozygous inactivation of HIF-2α in adult mice attenuates pulmonary hypertension and blunts right ventricular hypertrophy in response to chronic hypoxia [19]. The importance of HIF-2α in pulmonary hypertension is further evidenced in a mouse model of the human disease, Chuvash polycythemia. In this model, mice with a hypomorphic VHL reduces HIF-2α degradation and upregulates HIF-2α, thus leading to pulmonary hypertension [24]. The development of pulmonary hypertension in this model is partially rescued by homozygous inactivation of HIF-2α [24]. These studies reveal a direct involvement of HIF-2α in the development of pulmonary hypertension. However, the molecular mechanisms underlying the activation of HIF-2α, especially in vascular endothelium have not been clearly defined in pulmonary hypertension. Our recent study indicates that knockout of PHD2 increases EC/pericyte coverage *via* activation of the HIF-2α/Notch3 signaling pathway in the lung of LPS-treated mice [25]. Based on these findings, we hypothesize that specific knockout of PHD2 in EC would promote remodeling of pulmonary arterioles and lead to pulmonary hypertension by increasing pericyte coverage.

The present study examined the contribution of endothelial PHD2 signaling pathway in the development of pulmonary arterial hypertension using a novel endothelial-specific PHD2 knockout (PHD2^EC^KO) mouse that we developed. PHD2^EC^KO mice developed elevated pulmonary arterial pressure and right ventricular hypertrophy. The development of PAH was associated with remodeling of pulmonary arterioles, which was induced by HIF-2α activation, upregulation of Notch3/TGF-β and increased the pericyte coverage.

## RESULTS

### Development of pulmonary hypertension with a diastolic dysfunction in PHD2^EC^KO mice

We established an EC specific PHD2 knockout strain from mice by crossing a PHD2^f/f^ mouse with a Cdh5-Cre mouse reported to express Cre specifically in endothelial cells throughout the body. The results presented in Supplemental Figure 1 confirmed the loss of the PHD2 allele by tail DNA PCR analysis in mice. We also confirmed the loss of the expression of PHD2 protein in three independent EC lines isolated from PHD2^EC^KO mice (Supplemental Figure 2).

**Figure 1:**
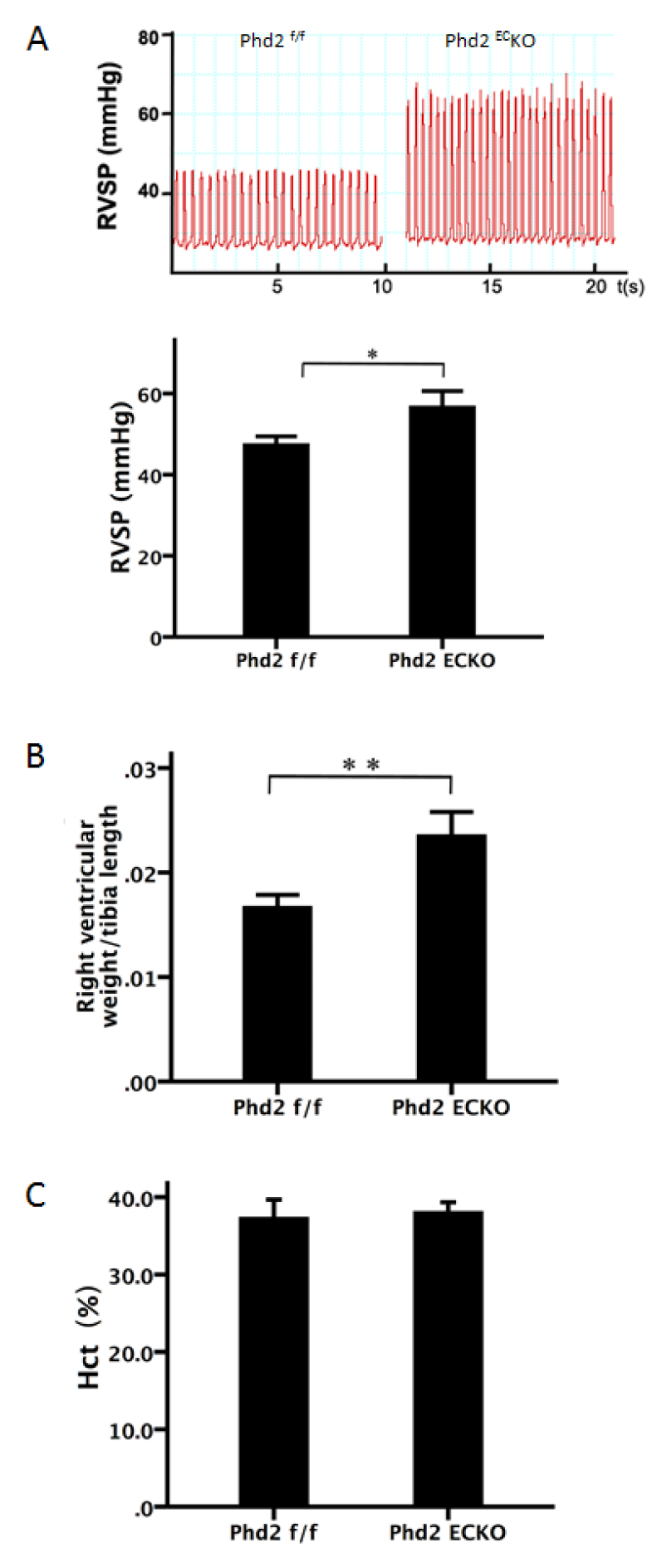
Development of pulmonary hypertension in PHD2KO mice **A.** The baseline hemodynamics of the pulmonary artery measurement showed that PHD2^EC^KO mice displayed elevation of right ventricular systolic pressure (RVSP). RVSP was significantly increased in PHD2^EC^KO mice compared with the PHD2^f/f^ mice (*n* = 8-9 mice). **B.** The ratio of right ventricular/tibia length was significantly increased in PHD2^EC^KO mice (*n* = 6-7 mice). **C.** No significant differences in the hematocrit (Hct) were found between PHD2^EC^KO mice and PHD2^f/f^ mice (*n* = 7-8 mice). Mean±SEM, *p < 0.05, **p < 0.01.

We used 15-month-old male PHD2^EC^ KO and control mice to determine the consequences of loss of endothelial PHD2. Body weight of the two groups were 38.8±2.1 g and 44.7±3.3 g, respectively. No significant difference between the two groups was found (*p* = 0.143). The measurement of baseline hemodynamics of the pulmonary artery revealed that PHD2^EC^KO mice had developed a pulmonary hypertension with a significant increase RVSP (Figure 1A). The right ventricular weight/tibia length was increased significantly in PHD2^EC^KO mice compared to PHD2^f/f^ control littermates (Figure 1B). There was no significant difference in the hematocrit between PHD2^EC^KO and PHD2^f/f^ mice (Figure 1C), suggesting that the elevation of RVSP following knockout of PHD2 in EC was not due to an increased number of red blood cells and polycythemia.

**Figure 2:**
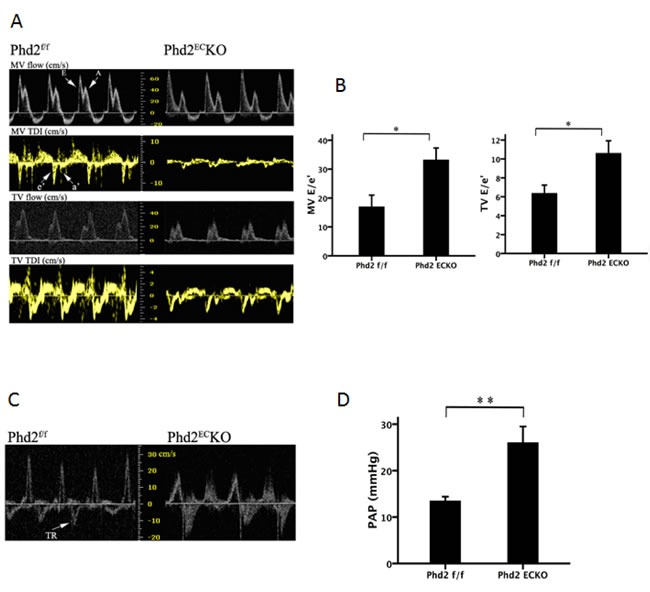
Right ventricular dysfunction in PHD2KO mice **A.** and **B.** The peak velocity of flow (E) and tissue (e') were measured by echocardiography. The ratio of peak velocity of flow (E) and tissue (e') is calculated. A significant difference in E/e' of MV and TV was found in the PHD2^f/f^ and PHD2^EC^KO mice. (*n* = 9-11 mice). **C.** and **D.** There was a significant difference in tricuspid regurgitation (TR) with higher pulmonary arterial pressure (PAP) in PHD2^EC^KO mice compared with the PHD2^f/f^ mice (*n* = 9-11 mice). Mean±SEM, *p < 0.05, **p < 0.01.

To further examine the functional consequences of endothelial PHD2 inactivation, we performed echocardiography. Transmitral inflow measured by Doppler ultrasound further revealed that the mitral valve E/e' ratio and tricuspid valve E/e' ratio were significantly increased, indicating both left and right ventricular diastolic dysfunction in the PHD2^EC^KO mice (Figure 2A and 2B). Moreover, knockout of PHD2 in EC significantly elevated pulmonary arterial pressure (PAP) compared to the PHD2^f/f^ mice (Figure 2C and 2D).

### Pulmonary arterial remodeling in the PHD2^EC^KO mice

**Figure 3:**
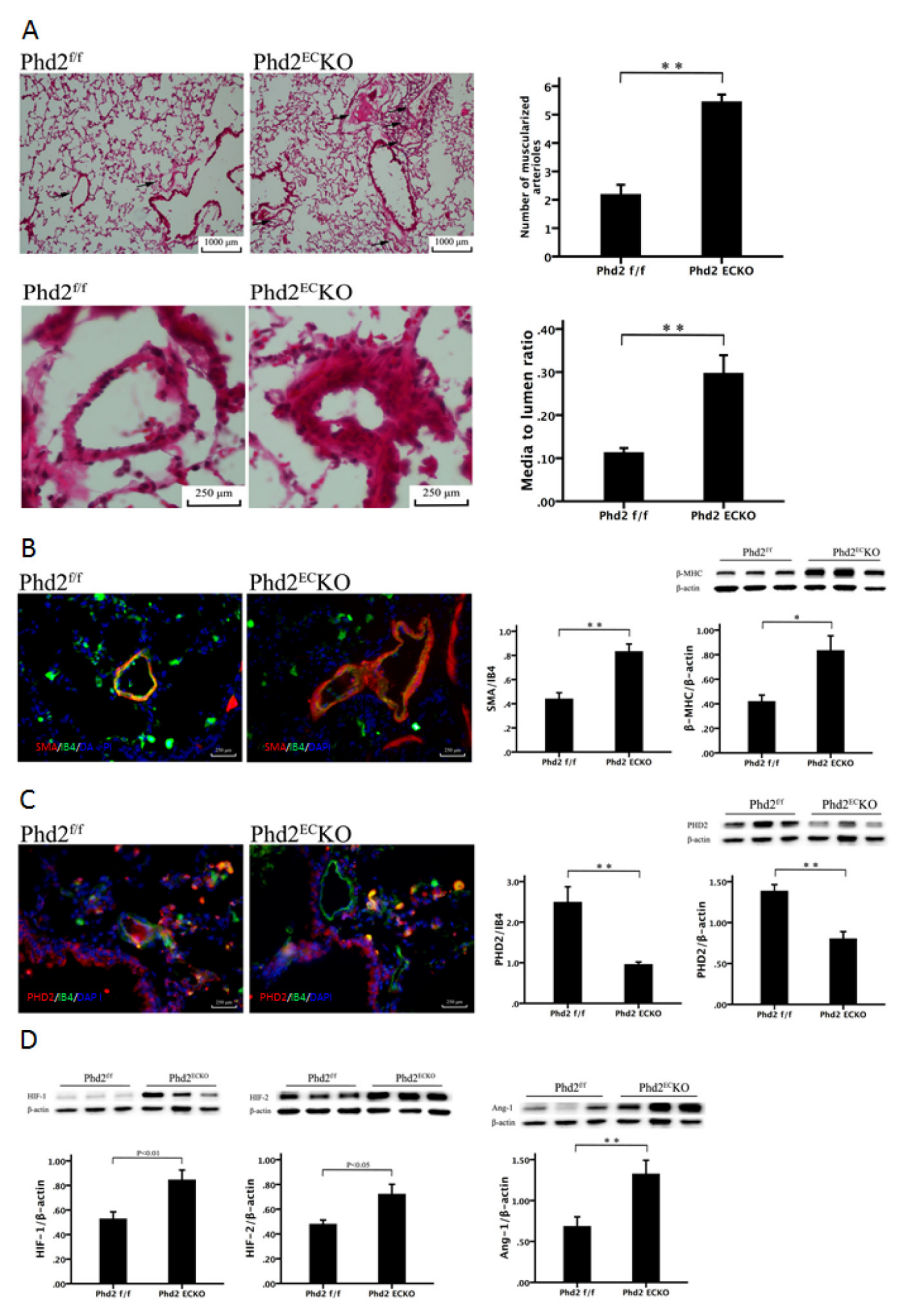
Deletion of PHD2 in EC caused pulmonary arterial remodeling in mice **A.** H & E staining showed that there was a significant increase in the number and thickness of small pulmonary arteries in the PHD2^EC^KO mice compared with that of the PHD2^f/f^ mice. Arrows show the small arteries. The number of muscularized arterioles (< 1,000 μm) was significantly increased in the lungs of the PHD2^f/f^ mice (*n* = 6 mice). The media to lumen ratio of pulmonary arteries was significantly increased in the PHD2^f/f^ mice (*n* = 6 mice). **B.** Immuno-histo-staining VSMC with SMA (red) and endothelial cells with IB4 (green) showed an increase in thickness of small pulmonary arteries in the PHD2^EC^KO mice. Area of SMA/IB4 (*n* = 3 mice) and the expression of β-MHC by western blot (*n* = 6-7 mice) were significantly increased in the PHD2^EC^KO mice. **C.** PHD2 (red) was absent in the endothelium (green). There was co-staining of EC and PHD2 (yellow) on the endothelium of PHD2^f/f^ mice, but not in the PHD2^EC^KO mice. The expression of PHD2 was significantly reduced in the lung tissue of PHD2^EC^KO mice, which was confirmed by both immuno-histo-staining (*n* = 5 mice) and western blot (*n* = 6-7 mice). **D.** Western blots analysis showed that the expression of HIF-1α, HIF-2α and Ang-1 was significantly increased in the PHD2^EC^KO mice (*n* = 6-7 mice). Mean±SEM,*p < 0.05, **p < 0.01.

As shown in Figure 3A, H&E staining indicated that the number of muscularized arterioles (< 1,000 μm) were dramatic increased in the lungs of PHD2^EC^KO mice. Moreover, the medial thickness of small pulmonary arteries (media/ to lumen ratio) was increased significantly compared with the controls (Figure 3A).

Lung sections were double stained with the EC marker, IB4, and the vascular smooth muscle cell marker, SMA, to assess pulmonary arterial remodeling. High-magnification images further confirmed these alterations, especially in small pulmonary arteries measuring < 1,000 μm. We therefore calculated the area of SMA/IB4 of pulmonary arteries of similar diameter (250-500 μm). As shown in Figure 3B, the area of SMA/IB4 staining was significantly increased in the PHD2^EC^KO mice. The expression of β-MHC protein that was associated with VSMCs proliferation, was significantly increased in the PHD2^EC^KO mice (Figure 3B). To confirm that PHD2 was absent in the endothelium, we double stained the lung tissue with PHD2 (red) and endothelial marker IB4 (green). In the PHD2^EC^KO mice, PHD2 positive cells were only seen in the perivascular region, but not in the endothelium of small pulmonary arteries. In the PHD2^f/f^ mice, PHD2 positive cells were observed in the vessel endothelium (yellow) (Figure 3C).

Western blot analysis further indicated there was a significant decline in the expression of PHD2 in the lung of PHD2^EC^KO mice (Figure 3C). This was accompanied by a significant increase in the expression of HIF-1α and HIF-2α. The expression of angiopoietin-1 was also significantly increased in the lung of PHD2^EC^KO mice (Figure 3D).

**Figure 4:**
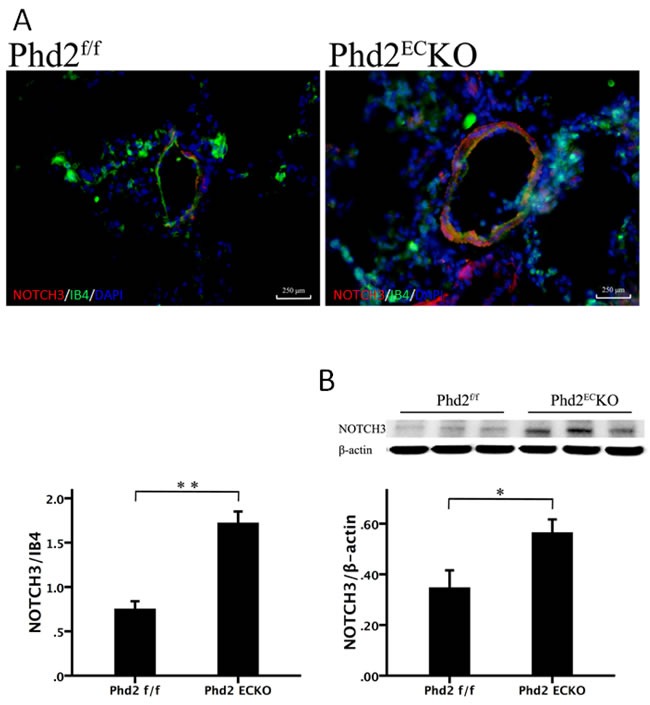
Deletion of PHD2 in EC upregulated Notch3 expression in mice **A.** The immunofluorescence staining showed that NOTCH3 (red) was mainly expressed in the vessel wall, which was double stained with NOTCH3 and IB4 (yellow). The area of NOTCH3/per vessel was significantly increased in PHD2^EC^KO mice compared with that of the PHD2^f/f^ mice. (*n* = 5 mice). **B.** Western blot analysis further revealed that the expression of Notch3 was increased in the PHD2^EC^KO mice (*n* = 6-7 mice). Mean±SEM, *p < 0.05, **p < 0.01.

Notch3 signaling is implicated in the control of smooth muscle cell proliferation leading to the development of PAH [26]. The immunofluorescence study revealed that Notch3 (red) was upregulated in the lung of PHD2^EC^KO mice compared to the controls (Figure 4A). Our western blot analysis further confirmed that the expression of Notch3 was upregulated in the lung of PHD2^EC^KO mice (Figure 4B). In addition, we found that Notch3 was mainly expressed in the vessel wall (IB4), suggesting that increased Notch3 may contribute to the medial thickness of small pulmonary arteries in the PHD2^EC^KO mice.

### Knockout of PHD2 in EC increases pericyte recruitment in pulmonary arteries

**Figure 5:**
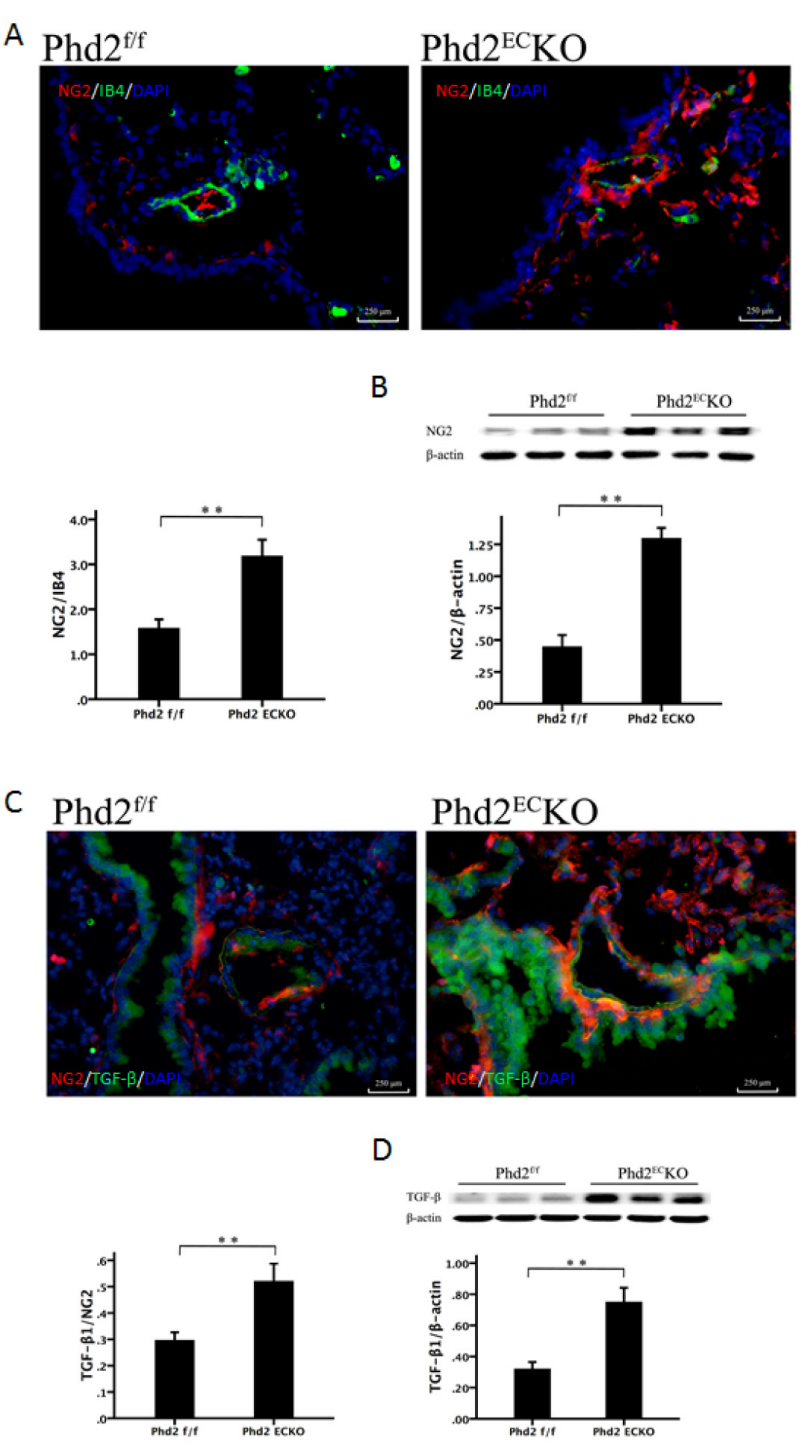
The recruitment of pericyte in the pulmonary arteriole **A.** The pericyte marker NG2 (red) stained positive cells were more abundant in the small pulmonary arteries of PHD2^EC^KO mice than that in the PHD2^f/f^ mice. The coverage area of NG2/IB4 was significantly higher (*n* = 5 mice). **B.** The western blot analysis revealed a significantly higher expression of NG2 protein in the PHD2^EC^KO mice (*n* = 6-7 mice). **C.** Immuno-staining revealed that the number of TGF-β^+^ (green) cells was increased in the small pulmonary arteries of PHD2^EC^KO mice. More TGF-β^+^/NG2^+^ cells (yellow) were found around the small pulmonary arteries of PHD2^EC^KO mice. The area of TGF-β^+^/NG2^+^ and the expression of TGF-β were increased significantly in the small pulmonary arteries of PHD2^EC^KO mice compared with that of the PHD2^f/f^ mice (*n* = 5 mice). **D.** The western blot analysis revealed that the expression of TGF-β was increased in the PHD2^EC^KO mice (*n* = 6-7 mice). Mean±SEM, **p < 0.01.

Since the recruitment of pericytes plays a critical role in the development of PAH, we examined the pericytes in the pulmonary artery. In the PHD2^EC^KO mice, there were more NG2 positive cells around the pulmonary artery and increased pericyte coverage (NG2/IB4) compared to PHD2^f/f^ mice (Figure 5A). Western blot analysis also indicated a significant increase in the expression of NG2 in the lung of PHD2^EC^KO mice (Figure 5B).

TGF-β has been reported to be upregulated in pulmonary hypertension and contribute to an increase in pericyte coverage [6]. In the present study, not only the number of TGF-β^+^ or NG2^+^ positive cells was significantly increased, but also more TGF-β^+^ and NG2^+^ double positive cells (yellow) were found in the lung of PHD2^EC^KO mice (Figure 5C). Similarly, the expression of TGF-β detected by western blot was significantly increased in the lungs of PHD2^EC^KO mice (Figure 5D), suggesting that pericytes may be recruited and differentiated into VSMCs *via* upregulation of the TGF-β signaling pathway.

### Knockout of PHD2 in EC increases pulmonary arterial perivascular fibrosis

**Figure 6:**
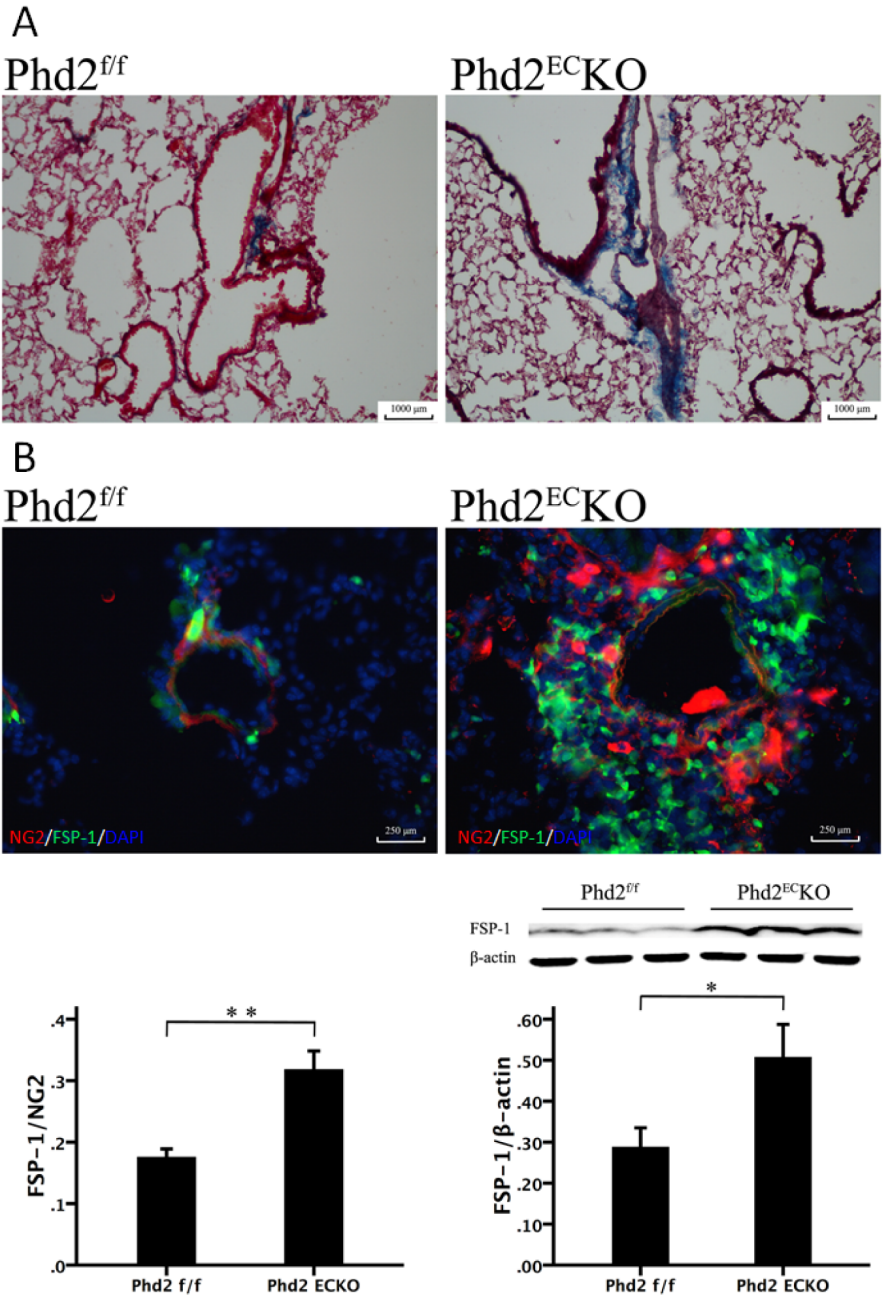
The Relationship between the Pericytes and fibrosis **A.** Masson trichrome staining showed that there was a dramatic increase in fibrosis formation (blue) in the small pulmonary arteries of PHD2^EC^KO mice. **B.** In the PHD2^EC^KO mice, both pericyte marker NG2 (red) and myo-fibroblast marker FSP-1 (green) stained cells were increased and scattered around the arteriolar. The area of FSP-1/NG2 measured by immunofluorescence was increased significantly in the small pulmonary arteries of PHD2^EC^KO mice compared with that of the PHD2^f/f^ mice (*n* = 4-5 mice). There was a significant increase of FSP-1 expression in the PHD2^EC^KO mice detected by western blot analysis. (*n* = 6-7 mice). Mean±SEM, *p < 0.05, **p < 0.01.

There was a dramatic increase in fibrosis formation in the small pulmonary arteries of PHD2^EC^KO mice (Figure 6A, blue) relative to age matched PHD2 floxed littermates. We further found that FSP-1, a marker of pericytes and differentiation of VSMCs to fibroblasts, was significantly increased in the PHD2^EC^KO mice detected by both western blot analysis and immunohistochemistry. The pulmonary vascular wall was double stained with NG2 (red) and FSP-1 (green) (Figure 6B). The co-staining study showed that the number of NG2^+^/FSP-1^+^ was significantly increased and scattered around the pulmonary arteries, indicating that pericytes may differentiate not only into VSMCs, but also into fibroblasts in the small pulmonary arteries (Figure 6B).

## DISCUSSION

In the present study, we found that specific deletion of PHD2 in the endothelium resulted in a significant rise in pulmonary arterial pressure together with right ventricular hypertrophy and dysfunction in mice. Furthermore, knockout of PHD2 in EC led to excessive pericyte coverage and perivascular fibrosis in pulmonary arteries. Mechanistically, knockout of PHD2 in EC upregulated Notch3/TGF-β levels that may increase pericyte coverage and promote differentiation into myofibroblasts. These results suggest an important role of endothelial PHD2 in the regulation of pericyte/VSMC recruitment and pulmonary vascular remodeling in PAH.

Pulmonary arterial remodeling is a hallmark of pulmonary hypertension [4, 5]. PAH is characterized by structural remodeling of small pulmonary arteries and arterioles, causing thickening of the vessel wall, and luminal occlusion by VSMCs and proliferation of endothelial cells [4, 5, 27]. During this progress, pulmonary vascular remodeling is one of the major contributors to the elevated pulmonary arterial pressure [28-31]. In PHD2^EC^KO mice, pulmonary hypertension and right ventricular hypertrophy were demonstrated. We speculated that this was due to the remodeling of pulmonary arteries, as evidenced by a significant increase the number of small muscularized arterioles and media/lumen ratio. The increased expression of β-MHC and SMA of pulmonary arteries in PHD2^EC^KO mice further supported the notion that PAH was associated with the proliferation of VSMCs. The perivascular interstitial fibrosis was also increased in the PHD2^EC^KO mice. These structural alterations may result in a reduction of compliance of small pulmonary arteries, which leads to right ventricular hypertrophy. Our echocardiograph revealed severe deterioration of diastolic dysfunction in PHD2^EC^KO mice. A previous study showed that global PHD2 conditional knockout mice developed severe polycythemia which contributes to heart failure [32]. Our study found a relatively normal hematocrit that excluded the possibility that the development of PAH and heart failure was mediated by erythropoiesis in PHD2^EC^KO mice.

Pericytes are vascular mural cells of mesenchymal origin, embedded in the basement membrane of the microvasculature, where they make specific local contacts with endothelium [33, 34]. For a long time, pericytes were thought to solely maintain capillary tone. However, recent genetic studies involving mouse mutants with reduced pericyte coverage of blood vessels indicate that pericytes actually have multiple effects on the vasculature. For example, capillary permeability is controlled by pericytes in the blood-brain barrier (BBB) [35, 36]. Detachment of pericytes from endothelial cells are associated with increased brain vascular permeability and impairment of BBB function [37]. In contrast, increased pericytes in distal pulmonary arteries, which function as a source of smooth muscle-like cell, causes pulmonary arterial medial thickening in human PAH and in rodent models of pulmonary hypertension [6]. Pericytes have also been shown to play a role in fundamental pathological processes including fibrosis, inflammation, thrombosis, and vessel calcification [38, 39]. HIF hydroxylase system plays a critical role in the regulation of vascular remodeling. In a recent study we showed that knockout of PHD2 prevents pericyte loss and increases pericyte/EC coverage in the lung of LPS treated mice [25]. However, the direct roles of endothelial PHD2 on pulmonary arterial remodeling and pericyte/EC coverage have not been previously investigated. In the present study, we found excessive pericyte coverage together with increased perivascular interstitial fibrosis in the PHD2^EC^KO mice. Although the origin of myofibroblasts in the lung remains controversial, three sources of precursor cells have been indicated: resident pericytes, mesenchymal stem cells, and alveolar epithelial cells [40-44]. Our data indicate that the number of NG2^+^ /FSP-1^+^ pericytes and FSP-1 levels were significantly increased in the pulmonary arteries of PHD2^EC^KO mice, suggesting that pulmonary arterial pericytes may differentiate into myofibroblasts. TGF-β is a ‘‘master switch'' in the differentiation of myofibroblasts in many tissues, including lung and heart. In the present study, we found that the number of NG2^+^ /TGF-β^+^ cells and the expression of TGF-β were significantly increased. These data provide a strong evidence that activation of TGF-β signaling pathways may contribute to pericyte differentiation into myofibroblasts in PHD2^EC^KO mice. Although the pulmonary vascular remodeling was evident at aged mice, the current study did not examine the early alterations of pulmonary vasculature in the PHD2^EC^KO mice. It is unknown at what time point these vascular remodeling occurred. A recent study revealed that knockout of PHD2 exhibited spontaneous severe PAH with extensive vascular remodeling at early age (3.5 months of age). These mice also exhibited progressive mortality, and higher death rate at 6 months of age [45]. In this study, Cre promoter was used to drive the deletion of PHD2 both in endothelial cells and hematopoietic cells (HCs) [45]. In our study, we had used endothelial specific VE-Cadherin-Cre (Cdh5-Cre) promoter to specific deletion of PHD2 in EC. Although very similar phenotype of pulmonary vascular remodeling was observed, the endothelial specific PHD2KO mice in our laboratory had a lower mortality and were able to survival after 15 months. Taken together, our study strongly suggests that the expression of PHD2 in endothelial cells plays a critical role in preventing pulmonary arterial remodeling.

Although accumulating evidence clearly supports the notion that pulmonary pericytes are potential source of VSMC-like cells that contribute to PAH pulmonary vascular remodeling [6], the underlying mechanisms that increase pericyte number and cause excessive pericyte coverage remain undefined. Notch3 expression is upregulated in human PAH [26]. Notch3 has been shown to regulate pericyte number and pericyte/EC coverage [46, 47]. However, little is known about Notch3 effects on pericyte density in pulmonary arterial remodeling. Our previous study suggests that PHD2 may regulate lung microvascular pericyte/EC coverage *via* upregulation of HIF-2α and Notch3 signaling pathways in the LPS-induced sepsis model [25]. Here, we found that specific deletion of PHD2 in EC upregulated HIF-2α and Notch3 in the pulmonary arteries. These results suggest the importance of Notch3 activation in the excessive pericyte coverage and VSMC proliferation of PAH. Ang-1, as a pericyte-derived paracrine mediator for the endothelium, is essential for pericyte recruitment and vessel maturation/stabilization. Our data show that Ang-1 levels were significantly increased in the lungs of PHD2^EC^KO mice. Overexpression of Ang-1 has been shown to promote pulmonary VSMC proliferation and is associated with the development of human PAH [48, 49]. Previously, we have shown that overexpression of Ang-1 increases VSMC *via* upregulation of Notch3 [50]. Taken together, these data implicate that Ang-1/Notch3 signaling pathway may be involved in the reciprocal communication between endothelial cells and pericytes. We speculate that deficiency of PHD2 in EC enhances EC-pericyte communication by increasing endothelial derived Ang-1 which modifies Notch3 and pericyte proliferation and recruitment, thus ultimately leads to excessive pericyte coverage in the pulmonary vasculature of PHD2^EC^KO mice.

In summary, our current work demonstrates a novel role of endothelial PHD2 in the pulmonary arterial remodeling of PAH. Deletion of PHD2 in the lung microvascular endothelium increased the number of pericytes, and promoted pericyte becoming myofibroblasts and VSMCs. The excessive pericyte coverage induced by the PHD2 deletion in the vascular endothelium was mediated by activation of HIF-2α/NOTCH3/TGF-β signaling pathway. The PHD2/HIF-2α pathway in the endothelium may be a novel candidate therapeutic target for chronic pulmonary diseases linked to PAH and pulmonary fibrosis.

## MATERIALS AND METHODS

### Generation of the PHD2^flox/flox (f/f)^ and PHD2^ECKO^ mice

PHD2^flox/flox^ (PHD2^f/f^) mice were obtained from Dr. Guo-hua Fong at University Connecticut. PHD2 ECKO mice were generated using the Cre-LoxP system as shown in Supplemental Figure 1. In brief, exon 2 of *Phd2* gene in PHD2^f/f^ mice was flanked with LoxP sites, for subsequent deletion by Cre recombinase. PHD2^f/f^ mice were crossbred with VE-Cadherin-Cre (Cdh5-Cre) transgenic mice [B6.FVB-Tg (Cdh5-cre) 7Mlia/J] obtained from Jackson Laboratories that express Cre recombinase under the control of the Cdh5 promoter in vascular endothelial cells. The resulting Cdh5-Cre/PHD2^flox/-^ heterozygous mutants were then mated with PHD2^f/f^ to obtain endothelial-specific ablated PHD2 mutant mice (PHD2^EC^KO) and PHD2^f/f^ mice. Experiments were performed on male mice at 15 months of age. Genotyping was performed by tail DNA PCR analysis. Tail DNA was obtained using Direct PCR (Tail) lysis buffer containing freshly prepared 0.5 mg/ml Proteinase K (VIAGEN Biotech, CA). Primer sequences used for genotyping the floxed *Phd2* allele were as follows: *Phd2* Forward 5′-CAA ATG GAG ATG GAA GAT GC-3′; and *Phd2* Reverse 5′-TCA ACT CGA GCT GGA AAC C-3′. The Cdh5-Cre transgene was detected using the following primers: transgene Forward 5′-GCG GTC TGG CAG TAA AAA CTA TC-3′ and transgene Reverse 5′-GTG AAA CAG CAT TGC TGT CAC TT-3′; internal positive control Forward 5′-CTA GGC CAC AGA ATT GAA AGA TCT-3′ and internal positive control Reverse 5′-GTA GGT GGA AAT TCT AGC ATC ATC C-3′. Primer sequences used for genotyping Phd2 ECKO were as follows: ECKO Forward 5′-AAC TCC GCC AAG CAG GTC AGA A-3′ and ECKO Reverse 5′-CCC GAA GAA CGA TAC CGT CGA G-3′. The PCR products were analyzed on 1.5% or 2% tris-acetate-EDTA (TAE) agarose gels stained with ethidium bromide (Supplemental Figure 1). To further confirm the deletion of PHD2 in vascular endothelium in the Cdh5-Cre/PHD2^flox/-^ heterozygous, lung microvascular endothelial cells were isolated and cultured. The expression of PHD2 and HIF-2α was examined by western blot analysis in these cells (Supplemental Figure 2).

#### Right ventricular systolic pressure (RVSP)

Experimental and sham control mice were anesthetized with ketamine (100 mg/kg) plus xylazine (15 mg/kg), intubated and artificially ventilated with room air. A 1.4-Fr pressure-conductance catheter (SPR-839, Millar Instrument, Houston, TX) was inserted into the right ventricle (RV) to record baseline hemodynamics of the pulmonary artery. The right ventricular systolic pressure (RVSP) was calculated and indexed as mmHg.

### Echocardiograph

Murine transthoracic echocardiography was performed with a Vevo 770 high-resolution micro-ultrasound system (Visualsonics Inc, Toronto, Canada). Flow through the mitral and tricuspid valves (E) and tricuspid regurgitation (TR) were measured by pulsed-wave Doppler. Tissue Doppler imaging (TDI) was used to detect the velocities of mitral and tricuspid annular movement (e'). E/e' ratio and pulmonary artery pressure (PAP) were calculated according to guideline recommendations [51, 52].

### Western blot analysis

Mouse lung tissues were homogenized with an ice-cold lysis buffer. The homogenates were centrifuged at 12,000 rpm for 15 minutes at 4°C and the total protein concentrations were determined using a BCA protein assay kit (Pierce Co, IL). An aliquot (30 μg) of the protein lysate was separated on a 10% SDS-PAGE gel and transferred to a polyvinylidene difluoride membrane by electrophoresis. The membranes were blocked with 5% nonfat dry milk in Tris-buffered saline and incubated with the following primary antibodies overnight: neural glial antigen (NG) 2, fibroblast specific protein (FSP) -1 (1:1000, abcam), PHD2, HIF-1α and HIF-2α (1:1000, Novus Bio, CO), Notch3, β-myosin heavy chain (MHC), transforming growth factor (TGF)-β and Ang-1 (1:1000, Sigma, MO). The membranes were then washed and incubated for 2 hours with an anti-rabbit or anti-mouse secondary antibody conjugated with horseradish peroxidase (1:5000, Santa Cruz, CA). Densitometric analysis of the bands were carried out using image acquisition and analysis software (TINA 2.0).

### Histological and immunofluorescence analysis

Lung tissues were fixed with buffered 10% formalin solution (SF93- 20; Fisher Scientific, Pittsburgh, PA), embedded in frozen optimal-cutting-temperature compound (4583; Sakura Finetek, Torrance, Calif) and 10μm frozen sections prepared. Some sections were stained with Hematoxylin & eosin (H & E). Pulmonary arterial media to lumen ratio and the number of muscularized arterioles (< 1,000 μm) were measured in 6 random microscopic fields. Some were directly immunostained with Alexa 488-stained Isolectin B4 (IB4) for endothelial cells, smooth muscle actin (SMA) for SMC and a NG2 antibody for pericytes (1:100, abcam). Other sections were immunostained with Notch3, PHD2, FSP-1 and TGF-β primary antibodies (1:200) followed by incubation with secondary antibodies conjugated with fluorescein isothiocyanate (FITC) or Cy3 (1:500). The area percentage of fluorescence was quantified by measuring 6 random microscopic fields containing at least one small pulmonary artery in the range of diameter from 250-500 μm using image-analysis software (Image J, NIH). Masson trichrome staining was also performed on adjacent sections to measure the degree of fibrosis.

### Statistical methods

Data are presented as mean ± SEM. The significance of differences in the means of corresponding values between groups were determined using the Student t test. *P* < 0.05 was considered to be significant.

## SUPPLEMENTARY MATERIAL FIGURES



## References

[1] Humbert M, Sitbon O, Chaouat A, Bertocchi M, Habib G, Gressin V, Yaici A, Weitzenblum E, Cordier JF, Chabot F, Dromer C, Pison C, Reynaud-Gaubert M (2010). Survival in patients with idiopathic, familial, and anorexigen-associated pulmonary arterial hypertension in the modern management era. Circulation.

[2] Morrell NW, Adnot S, Archer SL, Dupuis J, Jones PL, MacLean MR, McMurtry IF, Stenmark KR, Thistlethwaite PA, Weissmann N, Yuan JX, Weir EK (2009). Cellular and molecular basis of pulmonary arterial hypertension. J Am Coll Cardiol.

[3] Sacks RS, Remillard CV, Agange N, Auger WR, Thistlethwaite PA, Yuan JX (2006). Molecular biology of chronic thromboembolic pulmonary hypertension. Semin Thorac Cardiovasc Surg.

[4] Mandegar M, Fung YC, Huang W, Remillard CV, Rubin LJ, Yuan JX (2004). Cellular and molecular mechanisms of pulmonary vascular remodeling: role in the development of pulmonary hypertension. Microvasc Res.

[5] Voelkel NF, Gomez-Arroyo J, Abbate A, Bogaard HJ, Nicolls MR (2012). Pathobiology of pulmonary arterial hypertension and right ventricular failure. Eur Respir J.

[6] Ricard N, Tu L, Le HM, Huertas A, Phan C, Thuillet R, Sattler C, Fadel E, Seferian A, Montani D, Dorfmuller P, Humbert M, Guignabert C (2014). Increased pericyte coverage mediated by endothelial-derived fibroblast growth factor-2 and interleukin-6 is a source of smooth muscle-like cells in pulmonary hypertension. Circulation.

[7] Epstein AC, Gleadle JM, McNeill LA, Hewitson KS, O'Rourke J, Mole DR, Mukherji M, Metzen E, Wilson MI, Dhanda A, Tian YM, Masson N, Hamilton DL (2001). C. elegans EGL-9 and mammalian homologs define a family of dioxygenases that regulate HIF by prolyl hydroxylation. Cell.

[8] Ivan M, Kondo K, Yang H, Kim W, Valiando J, Ohh M, Salic A, Asara JM, Lane WS, Kaelin WG (2001). HIFalpha targeted for VHL-mediated destruction by proline hydroxylation: implications for O2 sensing. Science.

[9] Jaakkola P, Mole DR, Tian YM, Wilson MI, Gielbert J, Gaskell SJ, von KA, Hebestreit HF, Mukherji M, Schofield CJ, Maxwell PH, Pugh CW, Ratcliffe PJ (2001). Targeting of HIF-alpha to the von Hippel-Lindau ubiquitylation complex by O2-regulated prolyl hydroxylation. Science.

[10] Maxwell PH, Wiesener MS, Chang GW, Clifford SC, Vaux EC, Cockman ME, Wykoff CC, Pugh CW, Maher ER, Ratcliffe PJ (1999). The tumour suppressor protein VHL targets hypoxia-inducible factors for oxygen-dependent proteolysis. Nature.

[11] Bruick RK, McKnight SL (2001). A conserved family of prolyl-4-hydroxylases that modify HIF. Science.

[12] Ivan M, Haberberger T, Gervasi DC, Michelson KS, Gunzler V, Kondo K, Yang H, Sorokina I, Conaway RC, Conaway JW, Kaelin WG (2002). Biochemical purification and pharmacological inhibition of a mammalian prolyl hydroxylase acting on hypoxia-inducible factor. Proc Natl Acad Sci U S A.

[13] Taylor MS (2001). Characterization and comparative analysis of the EGLN gene family. Gene.

[14] Appelhoff RJ, Tian YM, Raval RR, Turley H, Harris AL, Pugh CW, Ratcliffe PJ, Gleadle JM (2004). Differential function of the prolyl hydroxylases PHD1, PHD2, and PHD3 in the regulation of hypoxia-inducible factor. J Biol Chem.

[15] Berra E, Benizri E, Ginouves A, Volmat V, Roux D, Pouyssegur J (2003). HIF prolyl-hydroxylase 2 is the key oxygen sensor setting low steady-state levels of HIF-1alpha in normoxia. EMBO J.

[16] Lieb ME, Menzies K, Moschella MC, Ni R, Taubman MB (2002). Mammalian EGLN genes have distinct patterns of mRNA expression and regulation. Biochem Cell Biol.

[17] Takeda K, Ho VC, Takeda H, Duan LJ, Nagy A, Fong GH (2006). Placental but not heart defects are associated with elevated hypoxia-inducible factor alpha levels in mice lacking prolyl hydroxylase domain protein 2. Mol Cell Biol.

[18] Tan Q, Kerestes H, Percy MJ, Pietrofesa R, Chen L, Khurana TS, Christofidou-Solomidou M, Lappin TR, Lee FS (2013). Erythrocytosis and pulmonary hypertension in a mouse model of human HIF2A gain of function mutation. J Biol Chem.

[19] Brusselmans K, Compernolle V, Tjwa M, Wiesener MS, Maxwell PH, Collen D, Carmeliet P (2003). Heterozygous deficiency of hypoxia-inducible factor-2alpha protects mice against pulmonary hypertension and right ventricular dysfunction during prolonged hypoxia. J Clin Invest.

[20] Bishop T, Ratcliffe PJ (2015). HIF hydroxylase pathways in cardiovascular physiology and medicine. Circ Res.

[21] Bigham AW, Lee FS (2014). Human high-altitude adaptation: forward genetics meets the HIF pathway. Genes Dev.

[22] Lorenzo FR, Huff C, Myllymaki M, Olenchock B, Swierczek S, Tashi T, Gordeuk V, Wuren T, Ri-Li G, McClain DA, Khan TM, Koul PA, Guchhait P (2014). A genetic mechanism for Tibetan high-altitude adaptation. Nat Genet.

[23] Song D, Li LS, Arsenault PR, Tan Q, Bigham AW, Heaton-Johnson KJ, Master SR, Lee FS (2014). Defective Tibetan PHD2 binding to p23 links high altitude adaption to altered oxygen sensing. J Biol Chem.

[24] Hickey MM, Richardson T, Wang T, Mosqueira M, Arguiri E, Yu H, Yu QC, Solomides CC, Morrisey EE, Khurana TS, Christofidou-Solomidou M, Simon MC (2010). The von Hippel-Lindau Chuvash mutation promotes pulmonary hypertension and fibrosis in mice. J Clin Invest.

[25] Zeng H, He X, Tuo QH, Liao DF, Zhang GQ, Chen JX (2016). LPS causes pericyte loss and microvascular dysfunction via disruption of Sirt3/angiopoietins/Tie-2 and HIF-2alpha/Notch3 pathways. Sci Rep.

[26] Li X, Zhang X, Leathers R, Makino A, Huang C, Parsa P, Macias J, Yuan JX, Jamieson SW, Thistlethwaite PA (2009). Notch3 signaling promotes the development of pulmonary arterial hypertension. Nat Med.

[27] Yuan JX, Rubin LJ (2005). Pathogenesis of pulmonary arterial hypertension: the need for multiple hits. Circulation.

[28] Eddahibi S, Humbert M, Fadel E, Raffestin B, Darmon M, Capron F, Simonneau G, Dartevelle P, Hamon M, Adnot S (2001). Serotonin transporter overexpression is responsible for pulmonary artery smooth muscle hyperplasia in primary pulmonary hypertension. J Clin Invest.

[29] Du L, Sullivan CC, Chu D, Cho AJ, Kido M, Wolf PL, Yuan JX, Deutsch R, Jamieson SW, Thistlethwaite PA (2003). Signaling molecules in nonfamilial pulmonary hypertension. N Engl J Med.

[30] Hansmann G, de Jesus Perez VA, Alastalo TP, Alvira CM, Guignabert C, Bekker JM, Schellong S, Urashima T, Wang L, Morrell NW, Rabinovitch M (2008). An antiproliferative BMP-2/PPARgamma/apoE axis in human and murine SMCs and its role in pulmonary hypertension. J Clin Invest.

[31] Thomas M, Docx C, Holmes AM, Beach S, Duggan N, England K, Leblanc C, Lebret C, Schindler F, Raza F, Walker C, Crosby A, Davies RJ (2009). Activin-like kinase 5 (ALK5) mediates abnormal proliferation of vascular smooth muscle cells from patients with familial pulmonary arterial hypertension and is involved in the progression of experimental pulmonary arterial hypertension induced by monocrotaline. Am J Pathol.

[32] Minamishima YA, Moslehi J, Bardeesy N, Cullen D, Bronson RT, Kaelin WG (2008). Somatic inactivation of the PHD2 prolyl hydroxylase causes polycythemia and congestive heart failure. Blood.

[33] Gaengel K, Genove G, Armulik A, Betsholtz C (2009). Endothelial-mural cell signaling in vascular development and angiogenesis. Arterioscler Thromb Vasc Biol.

[34] Armulik A, Abramsson A, Betsholtz C (2005). Endothelial/pericyte interactions. Circ Res.

[35] Armulik A, Mae M, Betsholtz C (2011). Pericytes and the blood-brain barrier: recent advances and implications for the delivery of CNS therapy. Ther Deliv.

[36] Armulik A, Genove G, Mae M, Nisancioglu MH, Wallgard E, Niaudet C, He L, Norlin J, Lindblom P, Strittmatter K, Johansson BR, Betsholtz C (2010). Pericytes regulate the blood-brain barrier. Nature.

[37] Nishioku T, Dohgu S, Takata F, Eto T, Ishikawa N, Kodama KB, Nakagawa S, Yamauchi A, Kataoka Y (2009). Detachment of brain pericytes from the basal lamina is involved in disruption of the blood-brain barrier caused by lipopolysaccharide-induced sepsis in mice. Cell Mol Neurobiol.

[38] Nees S, Weiss DR, Juchem G (2013). Focus on cardiac pericytes. Pflugers Arch.

[39] Juchem G, Weiss DR, Knott M, Senftl A, Forch S, Fischlein T, Kreuzer E, Reichart B, Laufer S, Nees S (2012). Regulation of coronary venular barrier function by blood borne inflammatory mediators and pharmacological tools: insights from novel microvascular wall models. Am J Physiol Heart Circ Physiol.

[40] Phan SH (2008). Biology of fibroblasts and myofibroblasts. Proc Am Thorac Soc.

[41] Kim KK, Wei Y, Szekeres C, Kugler MC, Wolters PJ, Hill ML, Frank JA, Brumwell AN, Wheeler SE, Kreidberg JA, Chapman HA (2009). Epithelial cell alpha3beta1 integrin links beta-catenin and Smad signaling to promote myofibroblast formation and pulmonary fibrosis. J Clin Invest.

[42] Hashimoto N, Jin H, Liu T, Chensue SW, Phan SH (2004). Bone marrow-derived progenitor cells in pulmonary fibrosis. J Clin Invest.

[43] Kalluri R, Neilson EG (2003). Epithelial-mesenchymal transition and its implications for fibrosis. J Clin Invest.

[44] Kisseleva T, Brenner DA (2008). Mechanisms of fibrogenesis. Exp Biol Med (Maywood).

[45] Dai Z, Li M, Wharton J, Zhu MM, Zhao YY (2016). Prolyl-4 Hydroxylase 2 (PHD2) Deficiency in Endothelial Cells and Hematopoietic Cells Induces Obliterative Vascular Remodeling and Severe Pulmonary Arterial Hypertension in Mice and Humans Through Hypoxia-Inducible Factor-2alpha. Circulation.

[46] Gu X, Liu XY, Fagan A, Gonzalez-Toledo ME, Zhao LR (2012). Ultrastructural changes in cerebral capillary pericytes in aged Notch3 mutant transgenic mice. Ultrastruct Pathol.

[47] Wang Y, Pan L, Moens CB, Appel B (2014). Notch3 establishes brain vascular integrity by regulating pericyte number. Development.

[48] Chu D, Sullivan CC, Du L, Cho AJ, Kido M, Wolf PL, Weitzman MD, Jamieson SW, Thistlethwaite PA (2004). A new animal model for pulmonary hypertension based on the overexpression of a single gene, angiopoietin-1. Ann Thorac Surg.

[49] Sullivan CC, Du L, Chu D, Cho AJ, Kido M, Wolf PL, Jamieson SW, Thistlethwaite PA (2003). Induction of pulmonary hypertension by an angiopoietin 1/TIE2/serotonin pathway. Proc Natl Acad Sci U S A.

[50] Zeng H, Li L, Chen JX (2012). Overexpression of angiopoietin-1 increases CD133+/c-kit+ cells and reduces myocardial apoptosis in db/db mouse infarcted hearts. PLoS One.

[51] Nagueh SF, Appleton CP, Gillebert TC, Marino PN, Oh JK, Smiseth OA, Waggoner AD, Flachskampf FA, Pellikka PA, Evangelista A (2009). Recommendations for the evaluation of left ventricular diastolic function by echocardiography. J Am Soc Echocardiogr.

[52] Rudski LG, Lai WW, Afilalo J, Hua L, Handschumacher MD, Chandrasekaran K, Solomon SD, Louie EK, Schiller NB (2010). Guidelines for the echocardiographic assessment of the right heart in adults: a report from the American Society of Echocardiography endorsed by the European Association of Echocardiography, a registered branch of the European Society of Cardiology, and the Canadian Society of Echocardiography. J Am Soc Echocardiogr.

