# Supplementary material for: Loss of prolyl hydroxylase domain protein 2 in vascular endothelium increases pericyte coverage and promotes pulmonary arterial remodeling
Source: Oncotarget. 2016 Aug 24;7(37):58848–61. doi: 10.18632/oncotarget.11585 (PMC5312280; doi:10.18632/oncotarget.11585)
Supplement: Supplementary file 1 [file oncotarget-07-58848-s001.pdf]

Loss of prolyl hydroxylase domain protein 2 in vascular endothelium increases pericyte coverage and promotes pulmonary arterial remodeling

Supplementary Material

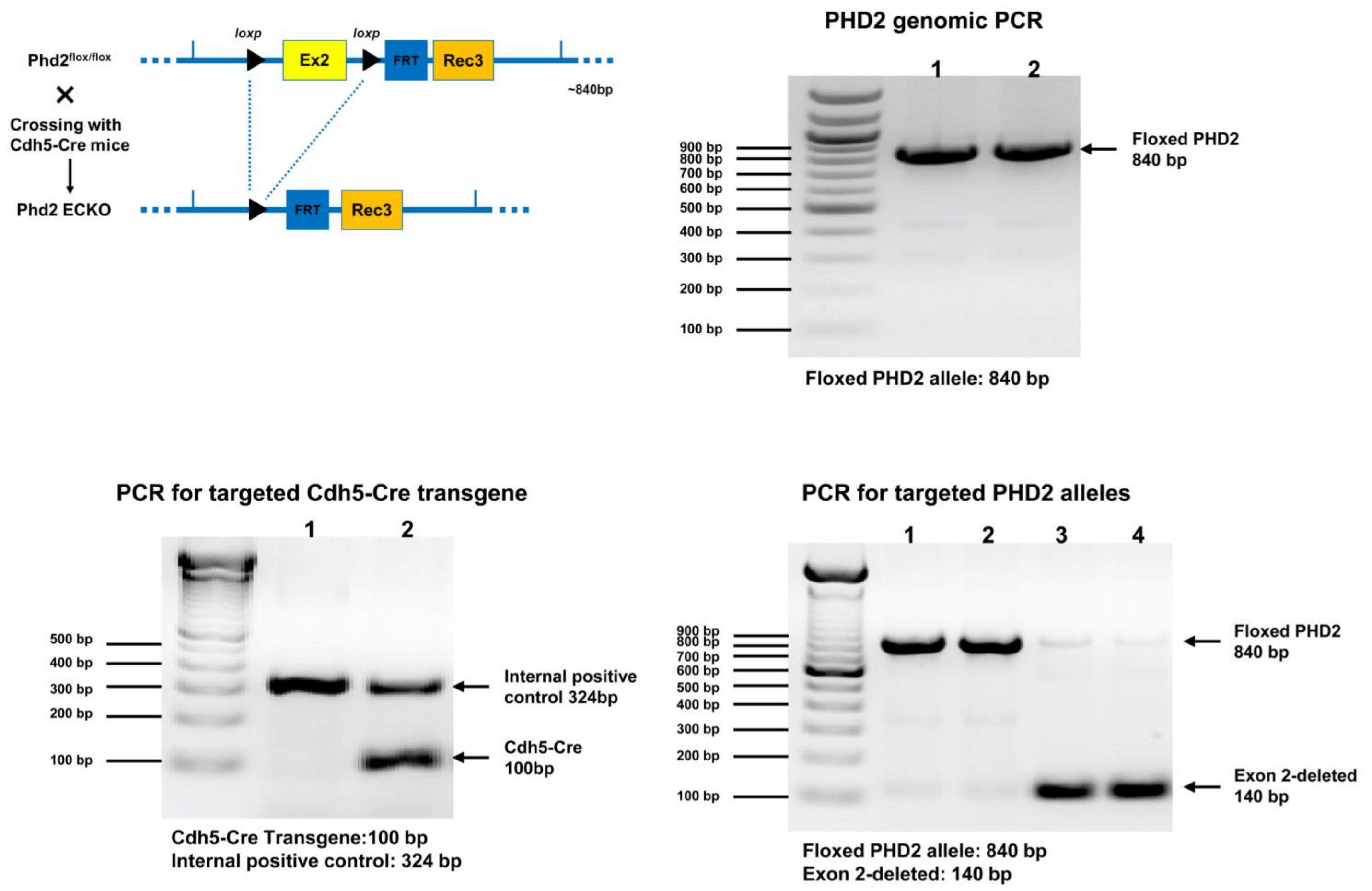

Supp Figure 1:

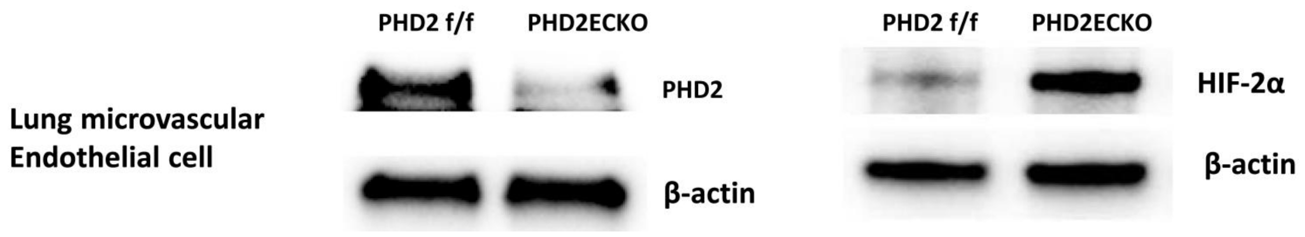

Supp Figure 2:
